# Combination of Supramicrosurgical Lymphatico-Venular Anastomosis (sLVA) and Lymph-Sparing Liposuction in Treating Cancer-Related Lymphedema: Rationale for a Regional One-Stage Approach

**DOI:** 10.3390/jcm13102872

**Published:** 2024-05-13

**Authors:** Guido Gabriele, Andrea Nigri, Glauco Chisci, Olindo Massarelli, Flavia Cascino, Ewa Komorowska-Timek, Kikuchi Kazuki, Hisako Hara, Makoto Mihara, Paolo Gennaro

**Affiliations:** 1 Department of Maxillo-Facial Surgery, Faculty of Medicine and Surgery, University of Siena, 53100 Siena, Italy; molindo74@gmail.com (O.M.); flaviacascino@hotmail.com (F.C.); paolo.gennaro@unisi.it (P.G.); 2 Department of Economics, Management and Territory, University of Foggia, 71122 Foggia, Italy; andrea.nigri88@gmail.com; 3 Oral Surgery School, Department of Medical Biotechnologies, University of Siena, Via Ricasoli 18, 58100 Grosseto, Italy; 4 Advanced Plastic Surgery, Michigan State University, East Lansing, MI 48502, USA; etimek@apsgr.com; 5 Department of Plastic Surgery, Nadogaya Hospital, 2-1-1 Shinkasiwa, Kashiwa 277-0084, Japan; kazuki560131@yahoo.co.jp (K.K.); hisakohara.prs@gmail.com (H.H.); mihara.plasticsurgery@gmail.com (M.M.)

**Keywords:** lymphatic, cancer, lymphedema, LVA, microsurgery, quality of life

## Abstract

**Objective:** Cancer-related lymphedema represents a potential complication of cancer treatment. The aim of this study is to evaluate the effectiveness of the combination of lymphatico-venular anastomosis and liposuction in the treatment of secondary lymphedema. **Methods:** We present a retrospective analysis of patients affected by cancer-related unilateral limb lymphedema. Inclusion criteria included previous neoplastic pathology with the consequent development of unilateral limb lymphedema, while the exclusion criteria included the presence of comorbidities and the persistence of cancer, as well as previous lymphatic surgery. The outcomes to be included were a reduction in the limb volume and lymphangitis rate, and an improvement in the quality of life. Patients’ data were assessed before surgery and 1 year after surgery. Perioperative management included clinical and ultrasonographical evaluations. Under local anesthesia, lymphatico-venular anastomosis with the supramicrosurgical technique and the liposuction of the affected limb was performed in the same surgical session. **Results:** A total of 24 patients were enrolled in the study. One year after the surgery, an average volume reduction of 37.9% was registered (*p* = 0.0000000596). The lymphangitis rate decreased after surgery from 4.67 to 0.95 per year (*p* = 0.000007899). The quality-of-life score improved from 68.7 to 16 according to the LLIS scale. **Conclusions:** The combination of LVA and liposuction represents a valid strategy for treating cancer-related lymphedema, ensuring stable results over time. In addition, it can be performed under local anesthesia, resulting in being minimally invasive and well-tolerated by patients. This paper reports on the short-term efficacy of this combined technique.

## 1. Introduction

Lymphedema is a complex progressive pathologic condition characterized by the accumulation of protein-rich fluid and the deposition of fibro-adipotic tissue in the subcutaneous layers. Lymphedema mostly occurs as a complication of oncologic treatments, in particular, lymphadenectomy and radiotherapy. The majority of data available regarding breast cancer show that the incidence varies from 15% to 54% four years after breast cancer treatment; the overall estimated incidence of this pathology was found to be 21.4%, affecting one in every five breast cancer patients [1,2,3].

In recent years, the understanding of the pathological processes accompanying lymphedema has substantially improved the therapeutic strategies in lymphedema management [4]. A growing body of evidence supports the effectiveness of modern surgical techniques, which can be categorized as physiologic, including lymphovenous bypass and lymph node transplant, or debulking by suction-assisted lipectomy [4,5,6]. Over time, both derivative and debulking treatments have been employed. In particular, the development of novel microsurgical techniques such as lymphatico-venular anastomosis or bypasses (LVA or LVB) has contributed to the amelioration of the prognosis of lymphedema patients [7]. Vascularized lymph node transfer (VLNT) involves the microvascular transplantation of functional lymph nodes into an extremity to restore physiological lymphatic function. The management of the advanced stages of lymphedema still remains difficult [8]. In fact, over time, chronic lymph stasis progressively alters the lymphatic vessels and regional tissues, causing fibrosis and fatty deposition in the affected area. Consequently, fibro-adipotic tissue is not responsive to derivative approaches nor to physiotherapy, and needs to be treated by adopting different strategies aiming to reduce excess tissue [9,10].

The majority of lymphedema patients who arrive to clinical observation and who manifest clinical complications show the intermediate stages of the condition, where a fluid component coexists with fibro-adipose tissue [1]. Among the most relevant complications, it is worth mentioning lymphangitis, i.e., inflammatory findings of the lymph vessels, clinically manifesting redness, tenderness, and/or heat, along the lymph vessels with or without limb swelling, and fever.

Upon careful clinical and instrumental examination, as well as in accordance with the results of histopathological studies, it has been shown that they have both a fluid component and an increase in fibro-adipose tissue. Therefore, to ensure effective treatment, it is appropriate to combine different techniques. Ultrasound and ICG lymphography have proved to be reliable to evaluate lymphedematous limbs, in particular regarding staging and function. Stage 1 and stage 2 patients show minimal or segmental dermal backflow, stage 3 patients show an intermediate condition, while stage 4 patients show a variable amount of dermal backflow involvement; finally, stage 5 patients show a diffuse pattern involving the entire limb. The combination of physiological approaches and debulking techniques on a regional basis appears to be effective and to offer long-lasting results [11]. Recently, some authors have reported some data regarding the possible role of liposuction and LVA in the treatment of lymphedema patients. Ciudad et al. reported good results in terms of the reduction in the limb circumference and infection rate with this combined technique [12]. Brazio and Nguyen also reported good results over 2.4 years with this combined technique [13].

In this manuscript, we present a case series of 24 patients affected by secondary lymphedema treated with the combination of LVA and lymph-sparing liposuction. The study aimed to evaluate the improvement of short- and long-term outcomes in patients with secondary lymphedema treated simultaneously with lymphatico-venular anastomosis and liposuction.

## 2. Materials and Methods

This is a retrospective observational study to evaluate the effectiveness of LVA and lymph-sparing liposuction in secondary lymphedema patients with a single limb affected. Treatment planning was discussed and the benefit/risk ratio was explicated to each patient, who subsequently signed a written informed consent form for participation in the present study, for the processing of personal data and images, and for publishing purposes. The study obtained approval by the Ethics committee (Comitato Etico Regionale per la sperimentazione Clinica della Regione Toscana, approval number 11048 2020), in accordance with the Helsinki Declaration for biomedical research involving human subjects and Good Clinical Practice Guidelines (General Assembly of the World Medical Association 2014).

Inclusion criteria included the following: previous neoplastic pathology which required the removal of lymph nodes, with the consequent development of secondary lymphedema; exclusion criteria included the presence of comorbidities or pathologies that precluded surgery and the persistence of neoplastic pathology, as well as previous lymphatic surgery. Outcomes to be assessed included the reduction in limb volume and in the lymphangitis number per year, as well as the improvement in quality of life as measured by the LLIS scale.

Patient demographics, history of lymphedema, objective findings, frequency of cellulitis, and hospitalizations were recorded preoperatively and 1 year after surgery, and were statistically analyzed. The lymphedema stage was evaluated according to the International Society of Lymphology. A quality-of-life questionnaire was proposed and patients were classified according to the stage of lymphedema. The study was reported in line with the STROCSS criteria [14].

### 2.1. Treatment

All patients underwent intensive physical therapy treatment one month before surgery. Patients were treated with elasto-compressive bandages, wherein they wore class II or III custom-made seamless compression garments, and manual lymphatic drainage sessions 3 times per week. Preoperative evaluation included clinical and photographical examination. Limb volume measurement was performed using the following truncated cone formula of both the affected and non-affected limbs: there were 2 circumferential measurements taken at opposite sides of the measured region and the volume of the limb was approximated by the truncated cone between them [14]. Moreover, quality of life was also assessed before and after treatment using the Lymphedema Life Impact Scale (LLIS). It consists of 18 questions distributed across physical, functional, and psychological domains and recorded on a 0-4 scale [15]. The quality of life in cancer patients represents a strong issue. In the recent literature, various lymphedema assessment tools have been reported, including the ULL-27 for upper limbs, the FLQA-L for arms and legs, and the WCLS in postmastectomy chronic disease. The LLIS scale is based on questions which cover the following four domains: symptoms, body image/appearance, function, and mood, and each item in each domain is scored between 1 and 4. In particular, patients complained of pain, the worsening of body image, and difficulty in wearing clothes [16].

All patients underwent preoperative ultrasonography of the affected limb using a linear multifrequence 14-4 mhz probe with the aim of identifying the areas of major fluid component from areas rich in fibro-adipotic tissue. Additionally, ICG lymphography was performed bilaterally with 3 subdermal injections of 0.1 mL of indocyanine green dye (Pulsion^®^) according to the existing literature [17,18]. This technique was employed to evaluate lymphedema using the Arm Dermal Backflow system [19].

**Figure 1:**
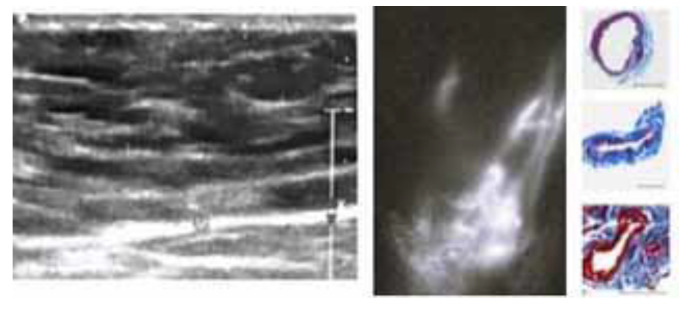
Instrumental and histological findings in the distal portion of an upper-limb stage III lymphedema. US demonstrates fluid accumulation with the consequent loss of subdermal pattern. ICG shows dermal backflow of a linear type with residual lymphatic function. Immunohistochemical examination with the lymphatic endothelial marker D2-40 (a monoclonal antibody to podoplanin) of a lymphatic vessel sample taken during the LVA shows lymphatics showing thin-walled vessels and patent lumen. These kinds of vessels still have contractility. The finding confirms that this area can receive effective LVA treatment.

**Figure 2:**
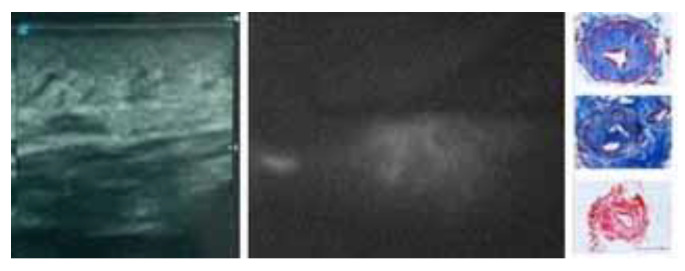
Instrumental and histological findings of the proximal portion of an upper-limb stage III lymphedema. US demonstrates fibro-adipose tissue with no fluid. ICG shows dermal backflow of the diffuse type with no apparent vessels. Even if the limit of depth of ICG lymphography and if lymphatics still persist and can be found, the overall residual lymphatic function is poor. Immunohistochemical examination with the lymphatic endothelial marker D2-40 (a monoclonal antibody to podoplanin) of a lymphatic vessel sample taken for scientific purposes for other studies shows a thick wall and restriction. These kinds of vessels have poor patency and contractility. The area should receive liposuction.

After the injections, lymphatic drainage was studied using an infrared camera system, the Photodynamic Eye (PDE), Hamamatsu Photonics, Hamamatsu, Japan. The number and quality of lymphatic collectors, lymphatic function, and dermal backflow patterns were analyzed, and the data were integrated with the ultrasonography results [20] (Figure 1 and Figure 2).

In each limb were identified both areas with residual lymphatic function, where the fluid component was predominant, and areas showing dermal backflow and the prevalence of fibro-adipose tissue. Consequently, the areas with the predominant fluid component and good lymphatic function were addressed with LVA, in most cases at the wrists and ankles, whereas the areas with major fibro-adipose tissue and poor lymphatic function received liposuction.

All surgeries were carried out under local anesthesia and light sedation. In accordance with the collected data and the preoperative mapping, the LVA and liposuction were performed in a single session. The LVA was performed by adopting the supramicrosurgical technique, as already described by the authors [21]. Multiple 2–3 cm cutaneous incisions (4 on average) were performed at the distal portion of a limb, in accordance with the lymphography findings. Supramicrosurgical anastomoses were practiced using 11/0 sutures in end-to-end, side-to-end, or octopus fashion according to the local vessels’ condition [22]. A milking test was performed at the completion of each anastomosis in order to verify its patency. Cutaneous incisions were closed with a non-resorbable 5/0 suture. Liposuction was performed after the completion of the LVA in the selected areas. The tumescent technique in a lymph-sparing fashion was adopted. Areas were infiltrated with a mix of a standard tumescent solution consisting of 1000 mL of saline solution, 50 mL lidocaine 1%, 1 mL epinephrine 1:1000, and 10 mL bicarbonate 8.4% for each liter. The infiltration volume was approximately from 0.5 to 1 L for the upper extremities and 1–2 L for the lower extremities. Two to four millimeter 3-hole blunt cannulas were employed. The aspiration technique was as parallel as possible along the lymphatic network pattern from the superficial to the deep layers. Areas where lymphatic vessels were previously identified at ICG lymphography, as well as the sites of LVAs, were spared. The volume of the aspirate was aimed to be approximately 80 percent of the volume difference between the affected and non-affected side in order to offset the increase in the limb volume due to the deposition of fibro-adipotic tissue in the subcutaneous layers, which cannot benefit from LVA treatment. Liposuction incisions were left open. The number and type of anastomoses were recorded as well as the amount of removed lipoaspirate.

Patients were discharged the day of the surgery; they received oral antibiotic and low-molecular heparin therapy for 1 week. Tight bandage compression was administrated 24/24 h for thirty days after surgery; then, they started physiotherapy again.

### 2.2. Follow-Up

Follow-up was scheduled at 1 month, 3 months, 6 months, and 1 year after surgery. Conservative postoperative treatments were carried out by specialized physiotherapists who followed discharged patients on a weekly basis; according to the LLIS scale, quality of life improved to 16. Moreover, after the first year, 1-year-after-surgery patient data were collected. Volume measurements and photos were taken, highlighting a significant reduction in lymphedema and a delay in physiotherapy treatment based on the evaluation of specialized personnel.

### 2.3. Data Analysis

We used the Shapiro–Wilk test, from which it emerged that the variables deviated from a hypothesis of normality (*p* < 0.05). As a result, we proceeded with a nonparametric approach using a Wilcoxon test for paired samples in order to compare the pre- vs. postmeasurements.

Then, we adopted the following statistical hypothesis system, where a *p* value of less than 0.05 was considered significant: *H*0:*μ*1 = *μ*2 {*H*1:*μ*1 > *μ*2.

## 3. Results

No postoperative major complication occurred. A total of 24 patients were enrolled in the study, including 22 females (92%) and 2 males (8%). They were free from cancer disease at the time of the study, but they had a history of neoplastic pathology which, regardless of the stage of the disease, had involved the surgical removal of lymph nodes resulting in the development of unilateral limb lymphedema. They presented with a history of mammary or gynecological cancer treated both with chemotherapy and surgery, and underwent periodical physiotherapy treatment, but did not present any disease which could interfere with the surgery.

The average age was 58± years old and ranged from 56 to 78 years old. The average duration of lymphedema was 5.66 years (range 2–8 years). A total of 20 (83%) were upper limbs, and 4 (17%) were lower limbs. All patients complained of recurrent lymphangitis; the average lymphangitis rate was 4.7 times per year. All patients were non-smokers. BMI was also calculated (Supplementary Materials).

**Figure 3:**
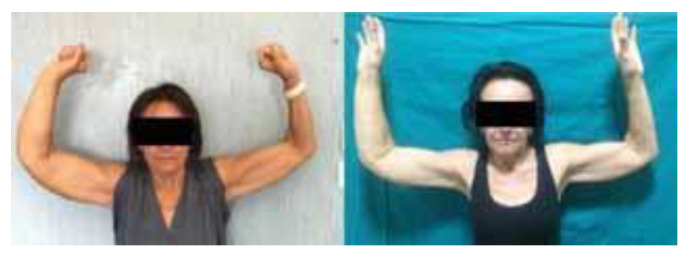
Patient affected by upper limb breast-cancer-related lymphedema. Preoperative and postoperative evaluation at 1 year. A volume reduction of 41% was measured.

**Figure 4:**
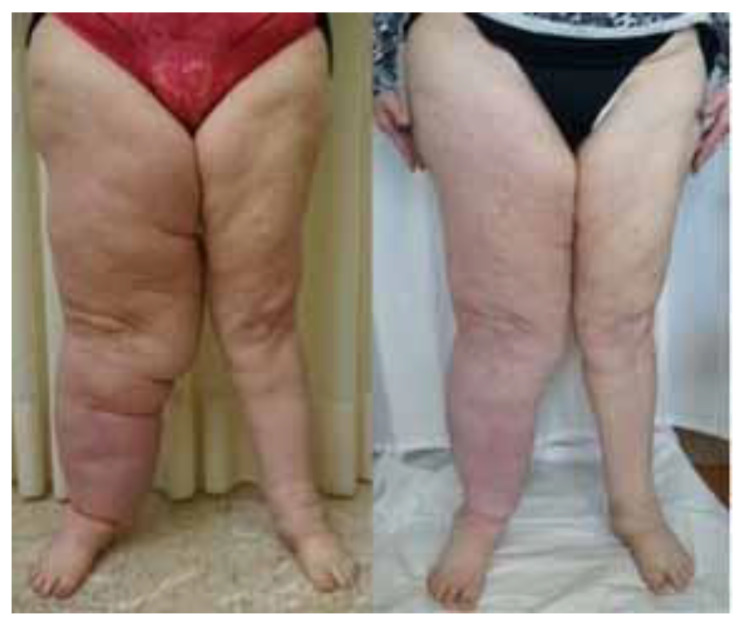
Patient affected by lower-limb cancer-related lymphedema. Preoperative and postoperative evaluation at 1 year. A 46% volume reduction was measured. The patient suffered from recurrent lymphangitis that significantly decreased after surgery.

All patients were affected by secondary lymphedema: 83% due to breast cancer, 8% due to ovarian cancer, and 8% due to uterine cancer. In particular, considering the stage according to the International Society of Limphology, 75% of patients presented stage III lymphedema, 20% presented with stage IV, and 4% presented with stage II. The average duration of the procedure was 170 min. The mean number of anastomoses was 5.5. The mean volume suctioned was 824 mL (500–1600 mL), in particular 773 mL (500–1000 mL) for the upper limbs and 1140 mL (1300–1600 mL) for the lower limbs (Figure 3 and Figure 4).

**Figure 5:**
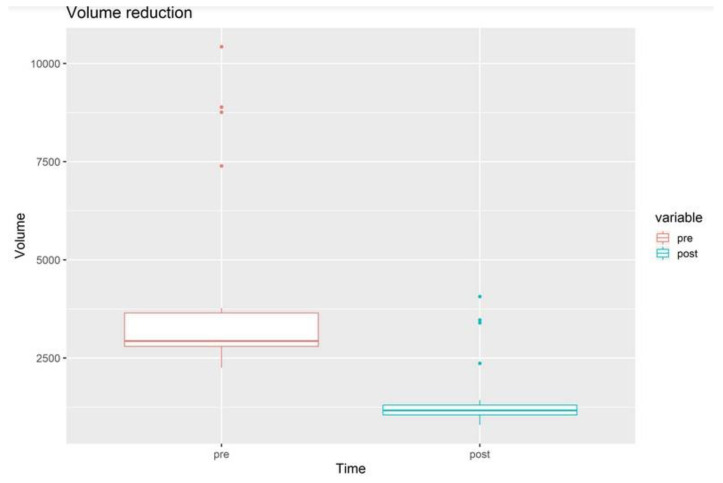
Statistical analysis of the volume measurements before and after surgery. Postoperative volume was significantly reduced. Average volume reduction was 37.9%.

**Figure 6:**
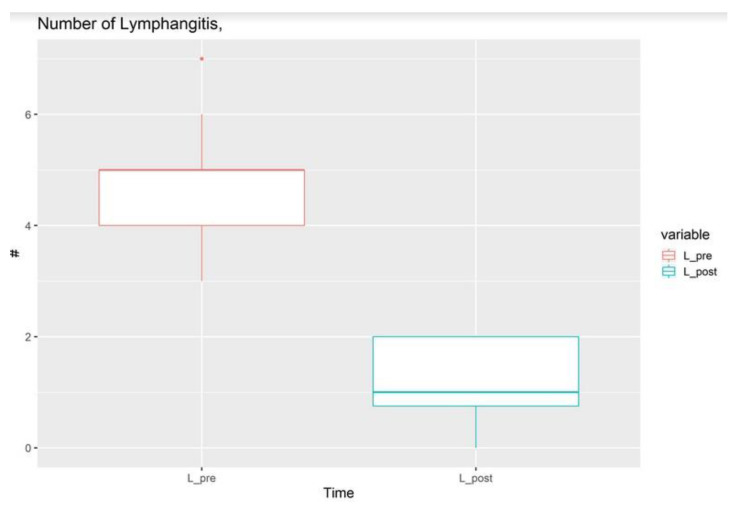
Statistical analysis of the lymphangitis rate before and after surgery. While before surgery an average of 4.6 lymphangitis episode was registered, after surgery lymphangitis reduced to 0.95 per year.

The average volume reduction was 37.9%, ranging from 27 to 51%. In particular, it was 27 to 51%, with a mean of 37.9% for the upper limbs, and 26% to 33% with an average of 30.2% for the lower limbs. (Figure 5) The lymphangitis rate after surgery reduced to 0.95 per year. (Figure 6).

The preoperative LLIS average score registered was 68.7. The postoperative quality-of-life index according to the LLIS was evaluated, demonstrating an average index of 16. In particular, patients appreciated the reduced sense of heaviness, improved body image, and ability to perform everyday duties and get dressed. Overall, significant differences (*p* = 0.0000000596) were found between pre- and postoperative volume measures. Regarding the groups of the number of lymphangitis cases, a significant difference (*p* = 0.000007899) was found between the pre- and postoperative conditions.

## 4. Discussion

Lymphedema is a progressive disabling condition characterized by the accumulation of interstitial fluid with subsequent inflammation, adipose tissue hypertrophy, and fibrosis [23]. Lymphedema and its complications have a heavy impact a patient’s quality of life, with high costs both for the healthcare systems and individuals [24]. According to the literature, there are approximately 1:1000 individuals affected by cancer-related lymphedema [25]. Chronic lymphatic insufficiency produces irreversible histological changes in the tissues of the affected areas. Overloaded lymphatic collectors progressively reduce their patency due to smooth-cell hyperplasia and the deposit of collagen; on the other hand, chronic inflammation leads to the accumulation of fibro-adipose tissue in the subcutaneous layers. Different factors have been identified as responsible for the adipose transformation in lymphedematous limbs. It has been shown that macrophages play an active role in adipocyte differentiation [26]. Furthermore, the interaction between inflammatory cells and the surrounding environment determines the upregulation of adipogenesis differentiating factors resulting in both hypertrophy as well as an increased number of adipocytes. In fact, the progression of lymphedema relates to the chronic evolution of the sclerotic and adipogenic mechanism [27]. Consequently, considering the nature of the pathology itself, on one hand, reducing lymph stasis and the fluid component will reduce the adipogenic mechanism; on the other hand, the removal of the para-physiological fat hypertrophy will downregulate the fluid overload and the chronic inflammation. Given the incidence and prevalence of this pathology, the identification of an effective surgical and conservative treatment is therefore of primary importance.

In this study, the majority of cases presented intermediate lymphedema stages, in particular stages III (75%) and IV (20%). These findings overlap with what lymphedema experts can observe in clinical practice worldwide. In fact, patients displaying lymphedema stage III to IV show complications which cannot be controlled only by conservative treatment or derivative surgical approaches. Derivative strategies appear to be more effective in the early stages, where the fluid component prevails in fibro-adipose tissue. In the intermediate stages of lymphedema, the overloaded fluid component is worsened by the accumulation of fibro-adipose tissue, therefore being eligible for the combination of LVA and liposuction. Scrupulous instrumental examinations are mandatory to evaluate the affected limb. ICG lymphography has been demonstrated to be reliable for several uses in surgery, especially for evaluating lymphatic flow both in lymphedema and in oncologic cases. Both ICG lymphography and ultrasonography are essential to gather information regarding the soft-tissue composition and lymphatic function. Both examinations should always be performed after having completed proper physiotherapeutic treatment [28]. ICG should be performed early after the injection with the aim of gathering more information regarding the lymphatic function. A lymphatic MRI can be employed to obtain both the identification of the lymphatic vessels and also to evaluate the composition of the subdermal compartments of the limbs [29]. In particular, in those areas where the extravasation of the lymph nodes and the loss of the dermo-epidermal junction were the prevalent features, liposuction appeared to be the elective choice.

Furthermore, an individual limb can present different stages of lymphedema. That leads to the necessity of a regional approach [30]. Different techniques can be combined in the same surgical session in order to address areas with different characteristics. Moreover, it is important to underline that the degeneration of the lymphatic system runs from proximal to distal areas [15]. Therefore, over time, generally the distal portion of a limb maintains a greater number of functioning lymphatic vessels; the increased volume has been shown to have a predominantly fluid component, and less adipose tissue can be found. Both the US and ICG can confirm these findings [16].

The US evidences areas with a fluid component where the extravasation of the lymph nodes and the loss of the dermo-epidermal junction can be observed. Likewise, in some cases, lymphatic vessels can be seen even if the US does not provide information about the lymphatic function. In the mentioned areas, early lymphography frequently shows a linear pattern, so that lymphatics with a good residual function can be identified and subsequently anastomized [16]. Those findings can often be observed in the distal portion of a limb. Conversely, the early examination of areas rich in fibro-adipose tissue through ICG lymphography does not demonstrate a linear pattern. In the majority of cases, a stardust or diffuse pattern with overloaded lymphatics can be observed. These types of lymphatics lack sufficient function and LVA is not recommendable [15].

Additionally, intraoperative findings with the histological analysis of lymphatic vessels demonstrate residual patency and contractivity. For this reason, distal areas can be treated by adopting derivative strategies. According to our experience, LVA appears to be the first-choice option [28].

Conversely, the proximal portions of the limbs show a lower number of functioning lymphatics, and adipose tissue tends to accumulate. In these areas, lymphatic liposuction should be considered. Therefore, in all intermediate stages, we propose a combined technique in a single session using a regional approach. In fact, the combination of LVA and liposuction in a single stage is more effective than LVA or liposuction alone, or the adoption of the two techniques in two stages, since it simultaneously offers the benefits of both techniques. Moreover, the functionality of the Mascagni pathway should be evaluated and preserved. Amongst all the derivative microsurgical techniques, LVA has proved to be a reproducible, effective, and minimally invasive procedure [30] able to treat various lymphatic conditions [31]. Many authors have reported its efficacy, and solid evidence can be found in the international literature. Also, liposuction represents a safe and standardized procedure with a small risk of complications. According to the infiltration/aspiration ratio, it can be dry, wet, super-wet, or tumescent [32]. Liposuction in lymphedema was first described by Brorson [28]. Dry liposuction under tourniquet control has been standardly performed in lymphedema in general anesthesia and with lifelong use of compression garments [33]. Currently, with this standard technique, a lifelong compression is requested, while a tumescent regional approach combined with LVA allows for the reduction in compression garments and the overall amount of physiotherapy. Common experience indicates that, in healthy individuals, tumescent liposuction does not provoke lymphatic dysfunction. Usually, in the physiologic condition, liposuction causes minimal injuries to the subcutaneous lymphatic network, which usually heals by itself with no consequence. Hence, in patients with pre-existent lymphatic dysfunction, it is fundamental to adopt a technique which must tend to spare, as much as possible, the functioning lymphatic tissues in order to prevent any further lymphatic injury [31,32,33].

Overall, LVA combined with liposuction has been found to be effective in treating secondary lymphedema, with better outcomes compared with a step-by-step procedure [9,34,35]. Therefore, the employment of the tumescent technique, small-diameter blunt cannulas, and meticulous US and ICG control is advisable. According to our experience, no skin excision was necessary, since the skin excess is expected to resolve with spontaneous skin contraction. We have observed an average volume reduction of 37.9% (Figure 3 and Figure 4).

A mean aspirate volume of 734 mL for the upper limbs and 1425 mL for the lower limbs has been reported. In the intermediate stage, the combination of LVA and liposuction is more effective than LVA and liposuction alone because lymphedematous limbs show both fluid and fibroadipose components. In addition, improvements are also related to the reduction in BMI after liposuction. Not only has the relation between increased BMI as a risk factor for lymphedema been widely demonstrated, but also the immediate benefit in terms of the volume reduction in improving patients’ compliance. Moreover, by the reduction in the BMI after liposuction, the lymphatic output is also reduced. Similarly, a significant reduction in the lymphangitis rate has been reported. The lymphangitis rate decreased from 4.7 to 1 per year, leading to a reduction in hospitalizations, pharmacological treatments, and healthcare costs. This finding can be mainly related to the LVA and its efficacy in reducing lymph stasis [20,36]. In addition, acute recurrent infections seem to be unfavorable for lymphedema evolution [37,38].

Some authors suggest that pitting edema can be treated adequately only by using continuous compression therapy without any lymphatic-derivative procedure. That can be true in selected cases, but clinical experience shows how difficult it is to treat and obtain satisfactory and stable results in a large number of patients. Moreover, it should be considered that the accessibility to physiotherapy changes widely from one country to another, and often inside different areas of the same country.

The limitations of this study include that it was not possible to free patients from long-term physiotherapeutic treatment, and the fact that patients with concomitant diseases, including cancer, were excluded from recruitment, which represents a relevant percentage in clinical practice. The measurement of postoperative values and a careful analysis is fundamental for evaluating the success of the procedures described. Some clinical cases may present evident results numerically but not optimally from an iconographic or statistical point of view; for this reason, it is important to increase the number of patients and for the follow-up to have a more clearly homogeneous result, and to not have influences dictated by a single case. In the case of a non-optimal but numerically evident result, it is important to underline the role of bandages and physiotherapy to also increase these sporadic cases.

The aim of the surgical treatment is to reduce the amount of physiotherapy over time. In fact, one year after surgery, patients were encouraged to reduce the number of sessions as well as the compression class at the minimum in order to maintain the results obtained, which was in agreement with their physiotherapist. In addition, economic costs, time consumption, and patients’ compliance are issues to be considered, making the lifelong necessity for continuous compressive treatments impracticable for many patients and not realistic in many countries nowadays. So, performing LVA and liposuction in a single session has the advantage of offering an immediate effect on patients, since it allows for the reduction in the volume of the affected limb and the feeling of heaviness, and in problems with clothing, thus producing an improvement in the patients’ quality of life. The LLIS represents a reliable and valid measure of quality-of-life impairment caused by lymphedema. Reliability was confirmed through excellent internal consistency and test–retest reliability. Although limb volume represents a common outcome measure employed in the treatment of lymphedema, the literature demonstrates a weak correlation between the edema volume and the function or quality of life; in fact, Cheng et al. [39] reported a reduction in the above-knee circumference, body weight, and episodes of cellulitis, and an improvement in the quality of life after lymph node transfer and LVA in lymphoedema patients. Losco et al. [40] suggested that timely treatment and BMI reduction are relevant in order to decrease the number of episodes of cellulitis and improve the quality of life. Arm-related symptoms and physical limitations adversely impact on the quality of life more than arm swelling. Evaluating impairments associated with lymphedema represents an important feature of treatments [10,18]. The high incidence of cancer-related lymphedema, its considerable impact, and the number of patients underline the importance of adopting effective and reproducible strategies and monitoring lymphedema patients [14].

## 5. Conclusions

A combination of LVA and liposuction based on a regional approach offers an effective and reproducible one-stage strategy to treat cancer-related lymphedema. Patients showing intermediate stages of lymphedema, especially stages III-IV, can be eligible to receive this technique. A significant reduction in the volume of the affected limb, in the lymphangitis rate, as well as stable results over time were observed.

## Data Availability

The data presented in this study are available on request from the corresponding author. The data are not publicly available due to privacy.

## Supplementary Materials

The following supporting information can be downloaded at: https://www.mdpi.com/article/10.3390/jcm13102872/s1.
