# Supplementary material for: Combination of Supramicrosurgical Lymphatico-Venular Anastomosis (sLVA) and Lymph-Sparing Liposuction in Treating Cancer-Related Lymphedema: Rationale for a Regional One-Stage Approach
Source: J Clin Med. 2024 May 13;13(10):2872. doi: 10.3390/jcm13102872 (PMC11121812; doi:10.3390/jcm13102872)
Supplement: Supplementary file 1 [file jcm-13-02872-s001.zip › jcm-3005069-supplementary.pdf]

Patients	Gender	Limb	Etiology	Stage	BMI	Affected period (years)	Pre-operative volume (ml)	Excess volume (ml)	Aspirate volume (ml)	Number of LVA	Volume reduction	Lymphangitis rate before surgery	Lymphangitis rate post surgery
1	F	Right arm	Breast cancer	III	28	7	2837	1106	780	4	39%	5	1
2	F	Left arm	Breast cancer	III	29	6	2943	1236	600	6	42%	4	2
3	M	Right arm	Breast cancer	IV	28	5	3624	1268	750	5	35%	6	0
4	F	Left arm	Breast cancer	III	27	8	2741	1042	600	7	38%	4	1
5	F	Right arm	Breast cancer	III	29	7	2831	906	900	5	32%	5	1
6	F	Right arm	Breast cancer	III	28	5	2256	812	500	6	36%	3	1
7	F	Left arm	Breast cancer	IV	29	6	3598	1223	800	4	34%	4	2
8	F	Right arm	Breast cancer	III	30	4	2731	1065	900	6	39%	6	2
9	F	Right arm	Breast cancer	III	29	6	2872	1063	750	6	37%	5	1
10	F	Right leg	Uterine cancer	IV	31	7	10427	4067	1600	5	39%	4	0
11	F	Right arm	Breast cancer	III	27	5	2952	800	600	6	27%	3	0
12	M	Left arm	Breast cancer	IV	28	5	3712	1299	800	4	35%	4	2
13	M	Right arm	Breast cancer	III	30	4	2773	1054	800	6	38%	5	1
14	F	Right arm	Breast cancer	III	33	2	2801	1429	500	6	51%	6	2
15	F	Left leg	Uterine cancer	III	29	7	8760	2365	1400	5	27%	5	1

16	F	Left arm	Breast cancer	III	27	5	2945	1090	1000	4	37%	4	1
17	F	Right arm	Breast cancer	III	32	8	2403	1009	800	6	42%	5	1
18	F	Left arm	Breast cancer	II	31	6	2866	1318	600	8	46%	4	2
19	F	Right arm	Breast cancer	III	30	4	2922	1110	600	6	38%	5	0
20	F	Left arm	Breast cancer	III	29	3	2749	1045	900	4	38%	6	0
21	F	Left leg	Ovarian cancer	IV	31	7	8890	3467	1400	6	39%	4	1
22	F	Right arm	Breast cancer	III	32	5	2996	1228	700	6	41%	5	1
23	F	Right leg	Ovarian cancer	III	33	4	7389	3399	1300	6	46%	7	2
24	F	Right arm	Breast cancer	III	28	6	3771	1282	800	4	34%	3	0
**average**					**29,5**	**5,5**	**3949,5**	**1486,4**	**849,2**	**5,5**	**37,9%**	**4,7**	**1,0**

Legend

Stage: lymphedema staging. Stage 1 and stage 2 patients show minimal or segmental dermal backflow, stage 3 patients had an intermediate condition, while stage 4 patients showed a variable amount of dermal backflow involvement; to end up, stage 5 patients had diffuse pattern involving the entire limb.

BMI: body mass index

LVA: lymphatico-venular anastomosis
